# Supplementary material for: Heterotrophy Compared to Photoautotrophy for Growth Characteristics and Pigment Compositions in Batch Cultures of Four Green Microalgae
Source: Plants (Basel). 2024 Apr 24;13(9):1182. doi: 10.3390/plants13091182 (PMC11085138; doi:10.3390/plants13091182)
Supplement: Supplementary file 1 [file plants-13-01182-s001.zip › plants-2859929-supplementary.pdf]

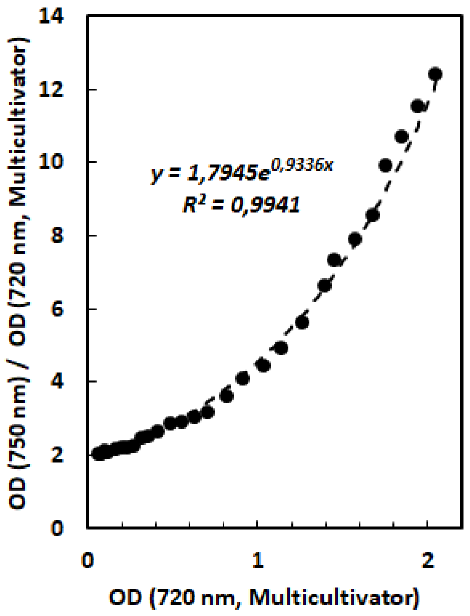


Figure S1. Example of fit of the relationship between the true OD (measured at 750 nm using a lambda 20 UV/Vis Perkin-Elmer Spectrophotometer) and the apparent OD720 signal provided by the Multi-cultivator. *Scenedesmus vacuolatus* cultivated in photoautotrophic conditions.

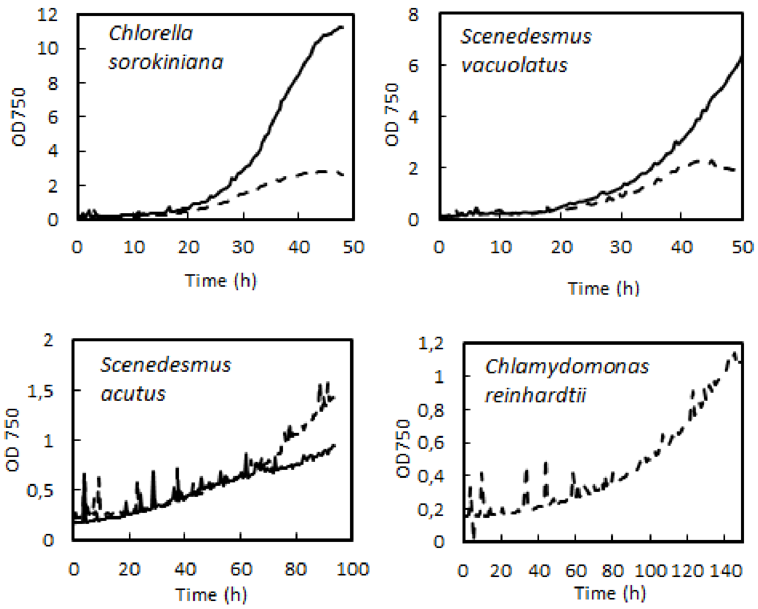


Figure S2. Typical OD heterotrophic growth curves on glucose 15 g.L$^{-1}$ (continuous lines) or acetate 2 g.L$^{-1}$ (dashed lines) in darkness.

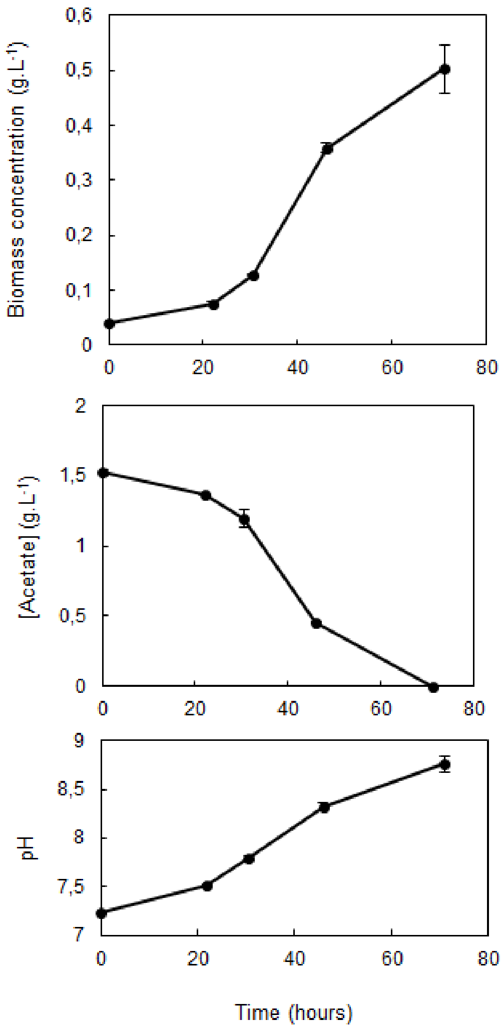


Figure S3. Acetate consumption and pH rise during heterotrophic growth of *Scenedesmus vacuolatus* on acetate (batch experiments conducted in well-aerated and agitated flasks at 25°C).
